# A scoping review of risk factors for urinary incontinence in older men

**DOI:** 10.1186/s12877-023-04249-7

**Published:** 2023-09-02

**Authors:** Olawunmi Olagundoye, Benjamin Odusanya, Janice Y. Kung, William Gibson, Adrian Wagg

**Affiliations:** 1 College of Health Sciences, Faculty of Medicine & Dentistry, Department of Medicine, Division of Geriatric Medicine, 1-198 Clinical Sciences Building 11350 – 83 Ave, Edmonton, AB T6G 2G3 Canada; 2 Manchester University NHS Foundation Trust, Manchester, UK; 3 John W. Scott Health Sciences Library, University of Alberta, Edmonton, AB Canada

**Keywords:** Aging, Older men, Risk factors, Urinary incontinence, Scoping review, Geriatrics

## Abstract

**Background:**

Most epidemiological studies have not systematically identified or categorized risk factors for urinary incontinence (UI) in older men, despite a higher prevalence than in younger men. Considering the burden of UI, an understanding of risk factors can inform cost-effective prevention/treatment programs. This scoping review aimed to identify and categorise risk factors for UI in older men, identify gaps in the evidence, and opportunities for future research.

**Methods:**

The Joanna Briggs Institute (JBI) method for scoping reviews guided the conduct and reporting of this review alongside the Preferred Reporting Items for Systematic Reviews and Meta-analyses extension for scoping reviews checklist. JBI’s Population, Concept, and Context approach framed the inclusion criteria (all evidence sources on UI risk factors that included older men [65 +]). We employed JBI’s three-step search strategy, which included a limited initial search in Ovid MEDLINE, a detailed comprehensive database search, and a search of reference lists of included studies, Google Scholar and grey literature. There were no restrictions on language, study type, or publication date. Two independent reviewers screened, selected, and extracted eligible studies. Data were analyzed using descriptive statistics and qualitative content analysis.

**Results:**

Forty-seven articles that met the inclusion criteria identified 98 risk factors across six categories. Behavioural risk factors, reported by only two studies, were the least investigated of all the categories, whereas medical factors/diseases were the most investigated. No genetic factors were documented. The top five risk factors were increasing age/advanced age (*n* = 12), Benign Prostatic Hyperplasia (*n* = 11), Diabetes Mellitus (*n* = 11), Detrusor overactivity (*n* = 10), limitation in physical function/ADL disability (*n* = 10), increased Body Mass Index (BMI)/overweight/obesity (*n* = 8), Dementia (*n* = 8), and Parkinson’s disease (*n* = 7).

**Conclusion:**

There is a dearth of evidence to describe the role behavioural risk factors have in UI in older men. These factors may play a role in health promotion and disease prevention in this area.

**Registration:**

A protocol detailing the methods was developed and published, and is registered in the Open Science Framework [Feb 07 2023; https://osf.io/xsrge/].

**Supplementary Information:**

The online version contains supplementary material available at 10.1186/s12877-023-04249-7.

## Background

The International Continence Society defines urinary incontinence (UI) as the complaint of any involuntary leakage of urine [1]. It affects both men and women of all ages, initially affecting more women than men, but this difference in prevalence decreases in association with increasing age. Moreover, one in three older men have problems maintaining continence [2]. Epidemiological studies suggest that UI prevalence among community-dwelling men ranges between 4.81% and 32.17%, and among older men (defined here as men 65 +) between 21% and 32% [3].

In Canada, UI costs to individuals, employers, and the health care system were calculated at $8.5 billion annually in 2014 [4]. The economic burden of UI in the United States has been estimated at more than $7000 (2009 USD) per individual per year, and it totals at least $39 million for male Medicare beneficiaries over 65 [5]. In 2001, US Census Bureau data estimated that approximately 3.4 million American men over the age of 60, either in the community or in nursing homes, suffered from UI, which was also associated with an increased risk of early death [6].

UI is under-reported and under-treated [6], particularly in older men, and there have been calls for more targeted research focusing on this specific group [7, 8]. A mindset that feminizes urinary incontinence has led to health inequalities and disparities in continence services for men [9], coupled with the fact that men are less likely to seek healthcare in general [10]. Although the impact of UI on health-related quality of life in older men and women is similar, most funded research has focused on women [11].

The limited research on male UI has mainly focused on its prevalence [3, 12, 13] and associated risk factors generally [3, 13, 14]. The majority of epidemiological studies of UI have neither systematically identified nor categorized risk factors for UI in older men.

In light of the medical, psychosocial, and financial burdens of UI, understanding risk factors can inform cost-effective prevention and treatment programs like self-management, a promising and proven intervention for managing chronic conditions [15]. Identifying the factors that can be modified will allow for the development of evidence-based interventions to help older men manage their own UI, a strategy previously found to be effective for women [16, 17]. Self-management intervention packages for men currently focus on uncomplicated lower urinary tract symptoms (LUTS) associated with prostate disease. Due to the heterogeneity of these recommendations [18–20], the lack of clarity regarding what might constitute an optimal self-management package, and the need to address the older population specifically [7], a scoping review of risk factors for UI in older men is necessary for a comprehensive mapping of the evidence [21].

### Urinary incontinence and risk factors

UI may be classified as potentially reversible or established [22]. Potentially reversible UI has a treatable cause and is more common among hospitalized older patients, and residents in long-term care [22] while established UI is chronic, and it may not be possible to identify a reversible cause. The five major types of established UI are urgency, stress (exertional), overflow, functional (disability associated), and mixed urinary incontinence [23].

Risk factors are characteristics, conditions, behaviours, or exposures that can increase the possibility of disease or injury [24]. Generally, risk factors can be grouped into categories: *Behavioural risk factors* relate to individuals’ actions, and can be eliminated or modified through lifestyle or behavioural changes [24]. *Physiological risk factors* are those relating to an individual’s body. They may be influenced by an interplay of genetics, lifestyle, and other broad factors. *Demographic risk factors* relate to the overall population. *Environmental risk factors* include social, economic, cultural, political, physical, chemical, and biological factors. *Genetic risk factors* are based on genetic makeup [24].

Although age groups were not specified, the Sixth International Consultation on Incontinence and the European Association of Urology document some established risk factors predisposing men to UI. They include increasing age, the presence of LUTS, urinary tract infections, functional and cognitive impairment, diabetes, alcohol intake, neurological disorders, and prostatectomy [8, 25]. The aetiology of UI, particularly in older adults, is multifactorial; risk factors coexist and interact to perpetuate the condition [24]. For example, Resnick described the case of an 80-year-old incontinent man whose evaluation confirmed the coexistence of multiple factors including Parkinson’s disease with limited mobility, congestive heart failure, and anticholinergic (haloperidol) use that caused faecal impaction and urinary retention and caused discomfort and confusion [22].

As part of a larger study, this scoping review aimed to synthesise evidence on risk factors as the starting point in the creation of a self-management intervention targeting older men [21].

### Objectives

This scoping review aimed to identify and categorise risk factors for UI in older men and identify gaps in the evidence. The overarching question addressed was “what are the risk factors for urinary incontinence in older men?”.

## Methods

This review was conducted in accordance with the Joanna Briggs Institute (JBI) method for scoping reviews [26], and reported in line with the Preferred Reporting Items for Systematic Reviews and Meta-analyses extension for scoping reviews (PRISMA-ScR) checklist [27]. A protocol detailing the methods was developed and published [21], and is registered in the Open Science Framework [Feb 07 2023; https://osf.io/xsrge/].

### Eligibility criteria

JBI’s Population, Concept, Context (PCC) framework, which highlights the relevant characteristics of the review’s participants (older men 65 +), the concept (UI risk factors), and refines the scope of the review by specifying a context (settings for older men), was used to develop the eligibility criteria. The PCC framework is detailed in the review protocol [21]. In brief, all data on UI risk factors stratified by age and sex, data on UI risk factors of 65 + males and females stratified by sex, male UI risk factors stratified by age, and data on UI risk factors of 65 + men only were included. The concept of risk factors for urinary incontinence was examined in all settings (community, acute care, post-acute care and continuing care). The sources eligible for inclusion were all study designs, including grey literature, without restrictions on publication date. For languages other than English, which comprised 10% of search results, we compared translations from two validated online language translators; DeepL translator and Google translator (https://www.deepl.com/en/translator and https://translate.google.com/).

### Search strategy

Following the JBI method, the three-step search strategy comprised an initial search in Ovid MEDLINE on May 24, 2022, a detailed search in all included databases on May 28, 2022, and lastly, a search of reference lists of included studies in February and March 2023. The medical librarian (JYK) developed and executed comprehensive searches over 4 h in Ovid MEDLINE, Ovid Embase, CINAHL, Scopus, Web of Science Core Collection, Cochrane Library (via Wiley), and ProQuest Dissertations & Theses Global. Keywords and controlled vocabulary were carefully selected to capture all relevant literature pertaining to risk factors for UI in older men. Appendix I (Additional file 1) describes full-text search strategies. Relevant studies published since the inception of the databases to the date of the detailed search were included. In addition to subscription databases, the research team reviewed the first 200 results from Google Scholar for inclusion. This is a reasonable number of results to screen since Web of Science and Google Scholar overlap heavily [28]. In the third step, bibliographies of the included studies were reviewed, as well as grey literature. When searching for grey literature in electronic format at different points in the review process, we used Google and websites of national and international organizations addressing the subject matter. All identified citations from the subscription databases were imported into Covidence (Veritas Health Innovation Ltd, Melbourne); a web-based collaboration software platform that streamlines the production of systematic and other literature reviews [29]. Following automatic removal of duplicates, two reviewers (OO and BO) independently screened all titles and abstracts identified with our literature search, after pilot-testing with a random sample of 5% of studies, which showed an almost perfect inter-reviewer agreement [30] (Cohen kappa coefficient; κ value) of 0.898. The Covidence database indicated moderate inter-reviewer reliability (κ value = 0.709) based on full text review. Potential reasons for exclusion were defined a priori, categorised, recorded, and reported in the scoping review. The full text of included citations was assessed in detail against the inclusion criteria by two independent reviewers (OO and BO). Conflicts detected by Covidence during the selection process were resolved through discussion and consensus.

### Data extraction

A customisable Covidence structure was used to develop the data extraction form (Appendix II/Additional file 1). Two reviewers (OO and AW) checked the draft extraction form through a calibration exercise to ensure the form captured all relevant data. The draft data extraction tool was modified and revised as necessary. Studies from Google Scholar and other sources were analysed and manually incorporated into the consensus data downloaded from Covidence. Table 1 summarizes the scoping review process and timelines.

**Table 1:** Tabular presentation of the scoping review process and timelines

**Stages**	**Actions**	**Timelines**
First step of the search strategy	An initial limited search in Ovid MEDLINE	May 24, 2022
Second step of the search strategy	A detailed comprehensive search in all included databases	May 28, 2022
Screening	Screening of titles and abstracts in Covidence and Google Scholar articles on Excel.	June – October 2022
Full text review	Review of full articles in Covidence and Google Scholar articles.	November – January 2023
Data extraction	[i] Extraction of data in Covidence [ii] Extraction of data from articles outside Covidence into the data master sheet after step 3 below.	January – March 2023
Third step of the search strategy	Search, screening and full article review of bibliographies of included articles and grey literature.	February – March 2023
Data analysis and report writing	Descriptive statistics, qualitative content analysis and manuscript preparation	March – April 2023

### Risk of bias

Following the JBI guidance, no quality appraisal was conducted, since the objective was to map the body of evidence without restriction in order to gain a deeper understanding and identify gaps, without testing hypotheses or trying to influence policy or practice [26].

### Data analysis and presentation

Using a predetermined framework, we extracted and analysed data deductively. The data were analysed qualitatively and quantitatively, using qualitative content analysis and descriptive statistics respectively. Results were stratified by the economic status of the country where the study was conducted, ethnicity/race, health context, inclusion criteria, types of UI and categories of risk factors. Tables, charts, and figures are employed to present quantitative data while qualitative data are organised into categories and presented as narrative summaries.

## Results/evidence synthesis

**Fig. 1:**
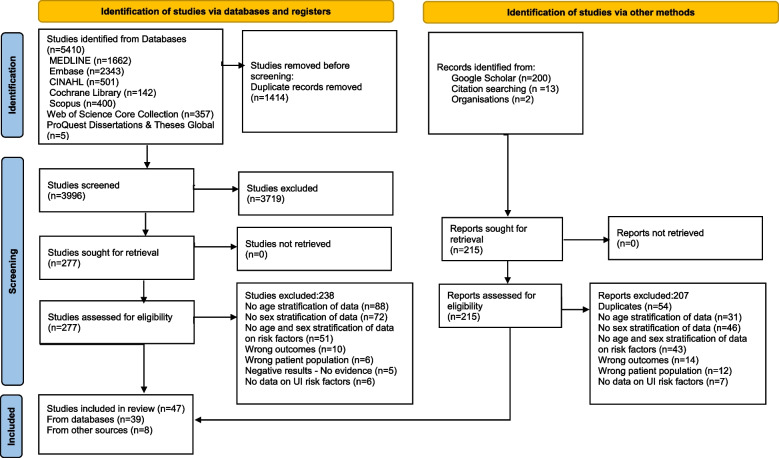
PRISMA flow chart

Forty-seven articles met the inclusion criteria for this review. Among the 491 eligible articles, 331 (67.4%) were excluded due to the lack of stratification of UI risk factors by age, sex, or age and sex, making them ineligible. Figure 1 shows the detailed selection process and exclusion reasons.

### Characteristics of the included studies

In Table 2, thirty-seven (79%) of the included articles were primary research articles. Of the 47 evidence sources, 21 (45%) were cross-sectional studies, 12 (26%) were cohort studies, 9 (19%) were review articles and the rest included an experimental study (2%), case series (*n* = 2, 4%), a report summary (2%) and a prevalence study (2%). Eighteen (38%) evidence sources were published in the past 10 years.

**Table 2:** Characteristics of the included studies

	Authors Year	Country in which the study was conducted	Study design	Total number of participants	Age	Population description	Behavioural risk factors	Physiological risk factors and age-related physiological changes	Demographic risk factors	Environmental factors	Medical factors/ diseases	Other factors
1	Bauer et al. 2019 [31]	United States	Cohort study	1,298	70—79	Men only	-	XXX	-	-	X	-
2	Zhang et al. 2021 [32]	China	Cross-sectional study	4,796	65 +	Men and women (sex-stratified risk factors data)	-	-	-	-	XXXXXXXXX	-
3	Nuotio et al. 2002 [33]	Finland	Cross-sectional study	NA	70 +	Men and women (sex-stratified risk factors data)	-	-	-	-	X	-
4	Palmer 1990 [34]	United States	Cohort study	434	65 +	Men and women (sex-stratified risk factors data)	-	-	-	-	X	-
5	Nazarko 1995 [35]	NA	Review article	NA	65 +	Men and women (sex-stratified risk factors data)	-	XXXXX	-	XXXXXXX	XXXXXX	XX
6	Maggi et al. 2001 [36]	Italy	Cross-sectional study	2,398	65 +	Men and women (sex-stratified risk factors data)	-	-	X	-	X	XX
7	Ostbye et al. 2002 [37]	Canada	Cohort study	826	65 +	Men and women (sex-stratified risk factors data)	-	-	X	X	XXX	X
8	Zunzunegui et al. 2003 [38]	Spain	Cross-sectional study	1,151	65 +	Men and women (sex-stratified risk factors data)	-	-	-	-	X	X
9	Lin et al. 2004 [39]	Taiwan	Case series	6	65 +	Men and women (sex-stratified risk factors data)	-	-	-	-	-	XXXX
10	Madersbacher et al. 2005 [40]	NA	Review article	NA	65 +	Men and women (sex-stratified risk factors data)	-	-	X	-	XXXXXX	XXX
11	Matsukawa et al. 2009 [41]	Japan	Cohort study	195	70 +	Men only	-	X	X	-	-	-
12	Wehrberger et al. 2011 [42]	Austria	Cross-sectional study	262	85 +	Men and women (sex-stratified risk factors data)	-	-	-	-	X	-
13	White et al. 2013 [43]	United States	Cross-sectional study	329,532	65 +	Male prostate and bladder Ca survivors and female Ca survivors	-	-	-	-	XX	-
14	McKibben et al. 2018 [44]	United States	Cohort study	113	66–79	Men undergoing AUS placement	-	-	-	-	-	X
15	Yang et al. 2019 [45]	United States	Cross-sectional study	10,826	65–79	Men and women (sex-stratified risk factors data)	-	-	-	-	XX	-
16	Cheng et al. 2020 [46]	Australia	Cross-sectional study	14,521	70 +	Healthy men and women	-	-	X	-	XX	-
17	Cheng et al. 2021 [47]	Australia	Cross-sectional study	19,114	70 +	Men and women (sex-stratified risk factors data)	-	-	-	-	X	-
18	Tse et al. 2021 [48]	China	Cross-sectional study	416	65 +	Men only	-	-	-	-	X	-
19	Prada et al. 2021 [49]	Romania	Cohort study	260	50 +	Men with BPH (age-stratified risk factors data)	XX	-	XX	-	XXXXX	-
20	Yamashita et al. 2022 [50]	Japan	Cohort study	121	65 +	Men post-robotic radical prostatectomy	-	-	-	-	-	XXX
21	Ouslander et al. 1981 [51]	NA	Review article		65 +	Men and women (sex-stratified risk factors data)	-	-	-	-	XXXXXXXXX	XXX
22	Murray et al. 1984 [52]	United States	Case series		74 and 78	Men and women (sex-stratified risk factors data)	-	-	-	-	X	-
23	Resnick et al. 1987 [53]	United States	Experimental study	32	68–94	Men and women (sex-stratified risk factors data)	-	-	-	-	-	X
24	Chan et al. 1992 [54]	Singapore	Cohort study	55	65–89	Men and women (sex-stratified risk factors data)	-	X	-	-	X	XXX
25	Anonymous 1995 [55]	NA	Report summary	NA	15 +	Adults and children (age and sex-stratified risk factors data)	-	-	X	XX	XX	X
26	Wetle et al. 1995 [56]	United States	Cross-sectional study	3,809	65 +	Men and women (sex-stratified risk factors data)	-	-	-	-	XXX	XXXX
27	Heath et al. 2002 [57]	NA	Review article		15 +	Men (age-stratified risk factors data)	-	-	-	X	X	XXXX
28	Landi et al. 2003 [58]	Italy	Cross-sectional study	5,372	65 +	Frail men and women (sex-stratified risk factors data)	-	-	X	XX	XX	XXX
29	Nuotio et al. 2003 [59]	Finland	Cross-sectional study	398	70 +	Men and women (sex-stratified risk factors data)	-	-	-	X	X	X
30	Kim et al. 2004 [60]	Japan	Cohort study	760	65 +	Men and women (sex-stratified risk factors data)	-	-	X	-	X	X
31	Boyington et al. 2007 [61]	United States	Prevalence study	95,911	65 +	Men and women (sex-stratified risk factors data)	-	-	X	-	-	-
32	Markland et al. 2008 [62]	United States	Cross-sectional study	1,000	65 +	Men and women (sex-stratified risk factors data)	-	-	-	-	XXXX	-
33	Goode et al. 2008 [63]	United States	Cohort study	496	65—106	Men and women (sex-stratified risk factors data)	-	-	-	-	-	XX
34	Chen et al. 2009 [64]	Taiwan	Cross-sectional study	594	65 +	Men only	-	-	-	-	X	XX
35	Gerst et al. 2011 [65]	United States	Cross-sectional study	796	75 +	Men only	-	-	X	-	XX	X
36	Kopp et al. 2013 [66]	United States	Cross-sectional study	5,990	65 +	Prostate Ca survivors	-	-	-	-	X	XXXX
37	Wang et al. 2017 [67]	Taiwan	Cross-sectional study	440	80 +	Men only	-	-	-	-	XX	X
38	Tsui et al. 2018 [68]	UK	Cross-sectional study	2,294	68 +	Men and women (sex-stratified risk factors data)	-	-	-	-	XX	-
39	Luo et al. 2022 [69]	China	Cross-sectional study	1,437	65 +	Men and women (sex-stratified risk factors data)	-	-	-	-	XXX	XX
40	Bauer et al. 2021 [70]	United States	Cross-sectional study	2,206	65 +	Men only	-	-	-	-	-	X
41	Hester et al. 2017 [71]	NA	Nonsystematic review	NA	65 +	Men only	-	-	-	-	XXXXXXX	XXXX
42	Griebling 2008 [72]	NA	Review article	NA	65 +	Men only	-	-	X	X	XXXXXXXX	XXX
43	Miller et al. 2011 [73]	NA	Review article	NA	65 +	Men with and without BPH	XX	XXX	X	XXX	XXXXXXXX	XXXXX
44	Moore 1999 [74]	NA	Review article	NA	65 +	Post-prostatectomy patients	-	-	X	-	XX	XXXXXXX
45	Neki 2016 [75]	NA	Review article	NA	65 +	Men and women (sex-stratified risk factors data)	-	-	-	-	XXXXX	-
46	Østbye et al. 2004 [76]	Canada	Cohort study	8,948	65 +	Men and women (sex-stratified risk factors data)	-	-	-	-	XXX	-
47	Hampson et al. 2021 [77]	United States	Cohort study	130	65 +	Men with SUI	-	-	-	-	X	XX

**Fig. 2:**
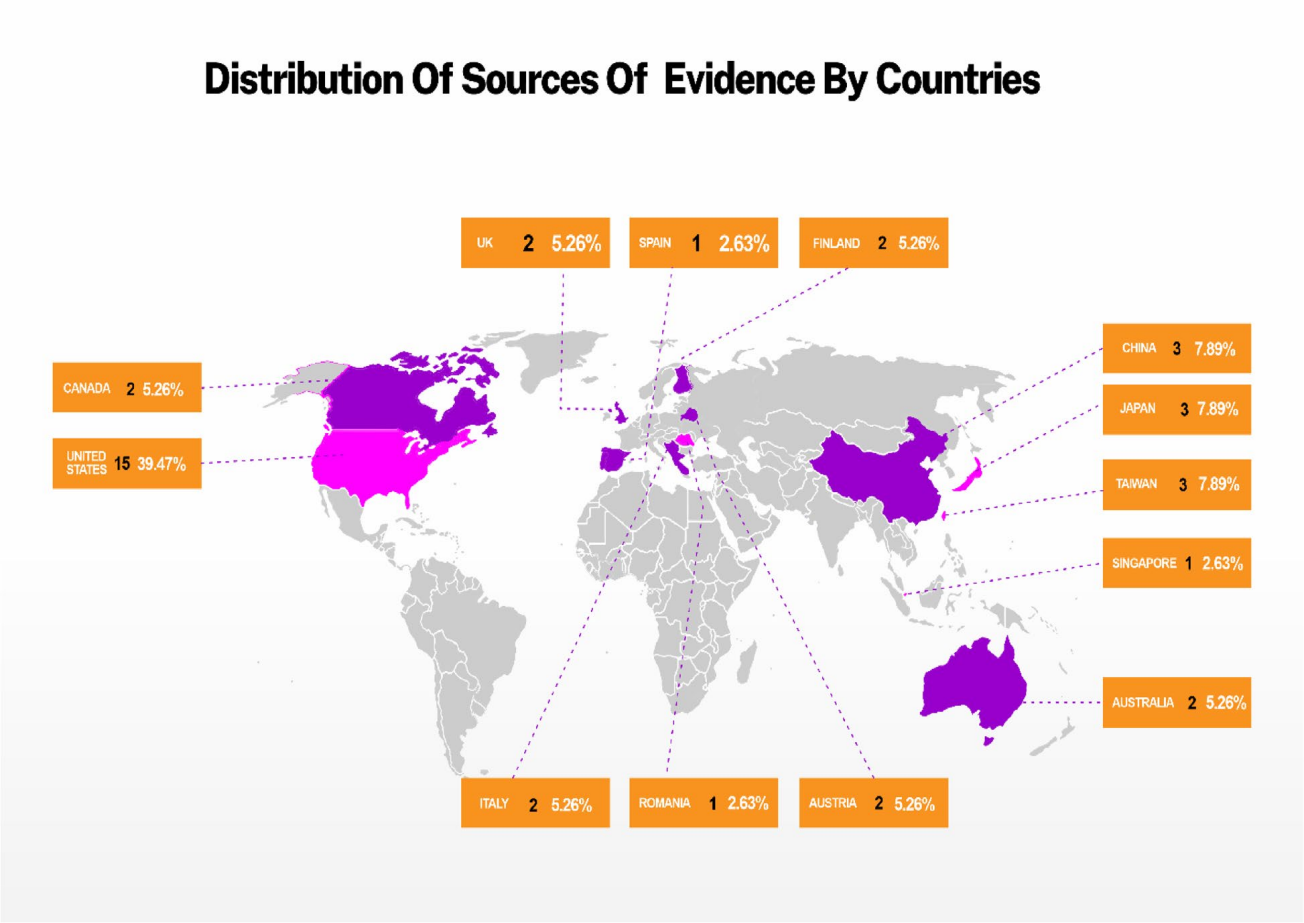
Map visualization showing evidence sources distribution by countries

Primary evidence sources were sparsely distributed across 12 of 195 countries. North America contributed almost half of the studies (*n* = 2, 5.3% from Canada and *n* = 15, 39.5% from the United States). Ten articles (26.3%) came from four Asian countries (China, Japan, Taiwan, and Singapore), and 10 articles (26.3%) came from six European countries (Austria, Finland, Italy, Romania, Spain, and the United Kingdom). None were from African and South American countries (Fig. 2).

**Fig. 3:**
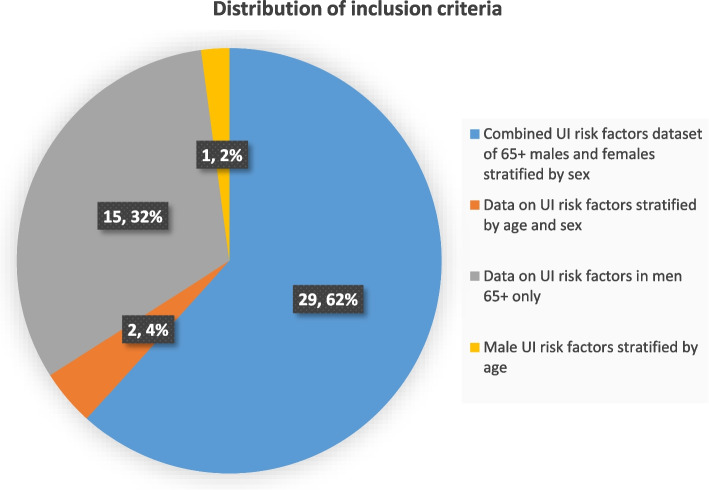
Distribution of inclusion criteria

Figure 3 shows that the majority (*n* = 29, 62%) of evidence sources were sex-stratified combined risk factor datasets for men and women 65 years and older, whereas one third focused solely on older men. Only a small percentage of included studies (*n* = 1, 2%) reported age-stratified data only in male samples.

**Fig. 4:**
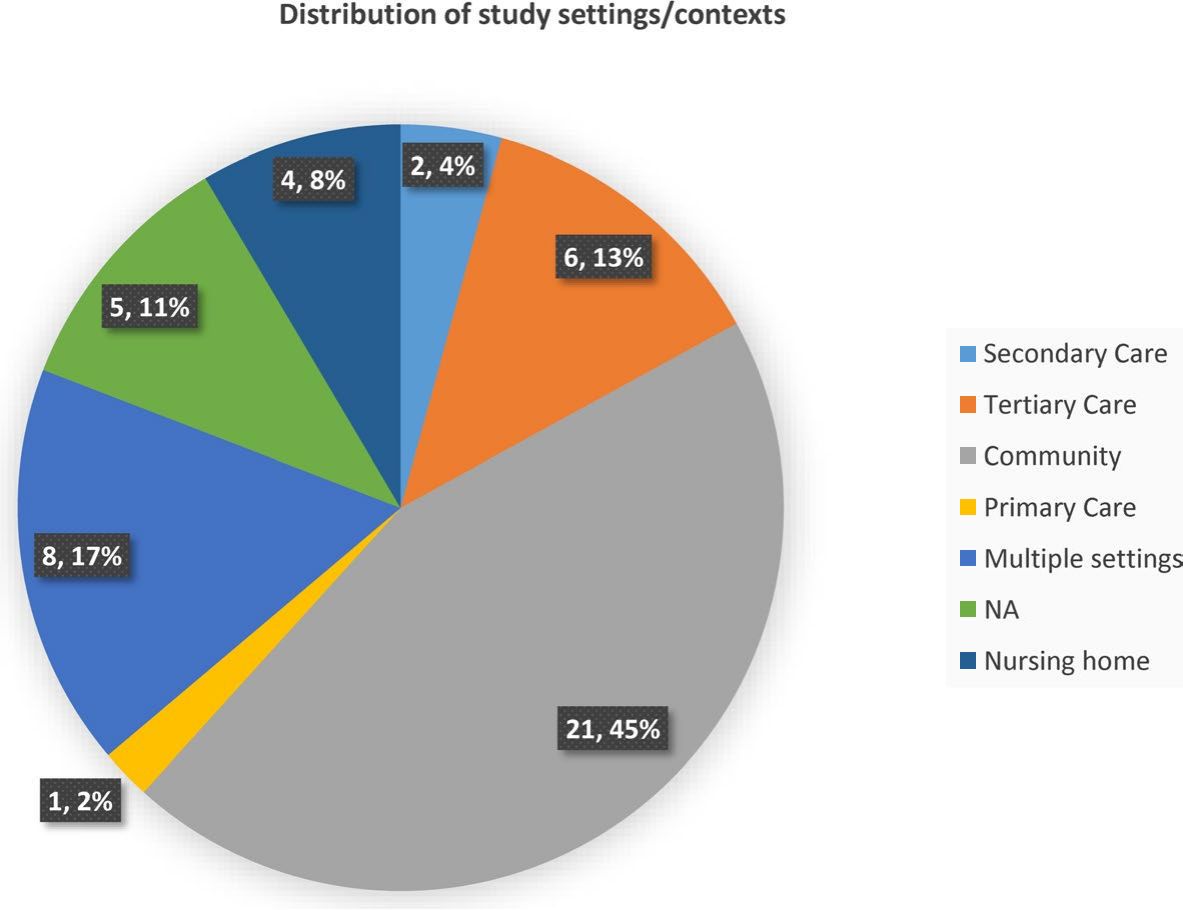
Distribution of study settings/contexts

Twenty-one (45%) evidence sources were community-based studies, while eight (17%) and six (13%) originated from samples from multiple settings and tertiary care facilities. Evidence from primary care settings was the least (*n* = 1, 2%) in Fig. 4.

### Risk factors for UI in older men

**Fig. 5:**
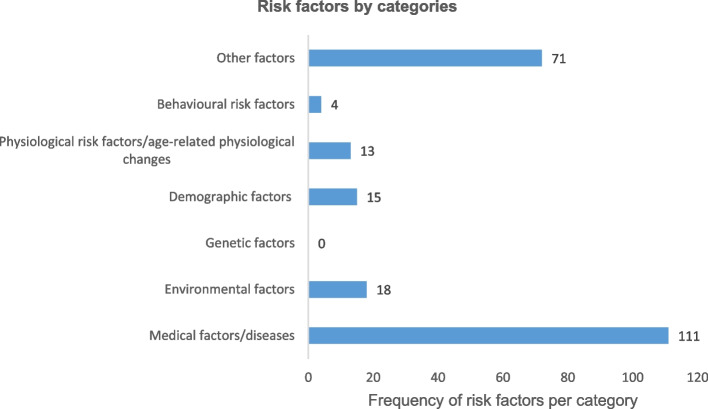
Frequency of risk factors by categories

Table 3 shows the details of 98 risk factors identified across six categories. A total of four behavioural risk factors, reported by only two studies, were the least investigated of all examined by these evidence sources, whereas 34 medical factors/diseases (with 111 frequency counts) were identified from 39 articles (83% of evidence sources). A total of 34 risk factors belonging to the other factors category were reported from 29 studies (62%) and were mostly medically-related entities that were not disease diagnoses. Nine physiological risk factors/age-related physiological changes were found in five studies (11%) with a frequency of 13. Four demographic factors with a frequency count of 15 were found in 14 studies (30%) and 13 environmental factors with a frequency of 18 were reported in eight studies (17%). Genetic factors were not documented. Figure 5 shows frequency counts across categories.

**Table 3:** Distribution of 98 risk factors by categories

Behavioral risk factors (4)	*n* = 4	Physiological risk factors and age-related physiological changes (9)	*n* = 13	Demographic risk factors (4)	*n* = 15	Environmental factors (13)	*n* = 18	Medical factors/ diseases (34)	*n* = 111	Other factors (34)	*n* = 71
Tobacco smoking	1	Increased fat mass	1	Age/Increasing age/Advanced age	12	Poor lighting	1	Increased BMI/overweight/ obesity	8	Detrusor overactivity/ Detrusor instability	10
Alcohol use	1	Greater waist circumference	1	Occupation	1	Cold	1	Heart disease	3	Limitation in physical function/ADL disability/low composite physical performance score	10
Caffeine intake	1	Decreased maximum grip strength	1	Residential area (urban dwelling)	1	Nursing home/ Institutionalization	4	BPH/Prostate problems	11	Immobility/Impaired mobility/unable to stand	2
Intake of bladder irritants (carbonated beverages, citrus, artificial sweeteners)	1	Decreased functional bladder capacity/Increased post void residual volume	3	Race/ethnicity	1	Lack of commode	1	Diabetes mellitus	11	Cognitive impairment	3
		Decline in renal function	1			Use of cot-sides/bedrails	1	Depression/ Depressive mood/symptoms	6	Hypnotics/sedatives	1
		Increase in nocturnal sodium and fluid excretion/Increased nighttime urine production	2			Early bedtime	1	Constipation/fecal impaction	3	Polyuria/Nocturnal polyuria	2
		Decreased awareness of bladder filling	1			Reliance on draw sheets and pads	1	Brain injury	1	Bladder outlet obstruction	4
		Decreased efficiency of bladder emptying	1			Call bells forgotten	1	Anxiety	1	Medications/ Polypharmacy	4
		Age-related declined urethral function	2			Sub-optimal nursing	1	Hypertension	2	Low serum testosterone	1
						Other environmental barriers	2	Voiding symptoms	1	Myosteatosis (Low average total psoas density)	1
						Physical restraints	2	UTI/Chronic UTI	6	Low obturator internus muscle thickness	1
						Commode at improper height	1	Stroke/Prior Stroke	6	Short membranous urethral length	2
						Bathroom distance	1	Parkinson’s disease	7	Prostatectomy	2
								Dementia	8	Anticholinergics	2
								Chronic diarrhea	2	DHIC	1
								Comorbid conditions/increased comorbidity	6	Atonic bladder	1
								Multiple sclerosis	3	Detrusor sphincter dyssynergia (DSD)	1
								Prostate cancer	3	Poor sleep quality/ sleep disturbance	2
								Bladder cancer	1	Self-perception of health	1
								Insomnia/sleep disorder	2	Frequency in getting out	1
								Heart Failure	2	Detrusor underactivity/ Underactive bladder	2
								Urethral stricture	1	Frailty	4
								Spinal cord compression/injury/ lesion	4	Incontinence less frequent than monthly	1
								Cervical spondylotic myelopathy	1	Post-radiation therapy/Preoperative radiotherapy	2
								Feacal incontinence	3	Androgen deprivation therapy for Prostate Ca (PCa)	1
								Chronic cough	1	Observation/watchful waiting in PCa	1
								Other chronic neurological diseases	1	Diuretics	1
								Poor vision	1	CNS depressant	1
								Sphincter injury	1	Alpha-antagonists and agonists	1
								Normal pressure hydrocephalus	1	Narcotic analgesics	1
								Delirium	1	Prostatectomy-related neural injury/ Pudendal nerve injury	1
								Hip fracture	1	Ischemia during surgery	1
								Kidney disease	1	Surgical technique	1
								Foot and ankle problems	1	Scar tissue immobilizing the sphincter	1

The top five risk factors were increasing age/advanced age (*n* = 12), Benign Prostatic Hyperplasia (*n* = 11), Diabetes Mellitus (*n* = 11), Detrusor overactivity (*n* = 10), limitation in physical function/ADL disability (*n* = 10), increased Body Mass Index (BMI)/overweight/obesity (*n* = 8), Dementia (*n* = 8), and Parkinson’s disease (*n* = 7).

For qualitative content analysis, findings are organised into categories according to the PCC framework.

### UI risk factors and contexts

Five of the 20 articles focusing exclusively on the community setting suggested that age was a significant demographic risk factor [36, 46, 58, 60, 65]. Five community-based studies documented urgency UI (UUI) as the most common established UI type [31, 32, 65, 67, 68], while stress UI (SUI) was less prevalent [42, 46, 47, 69]. Physiological factors associated with UUI included increased fat mass (participants’ mean total fat mass = 24 kg), greater waist circumference (mean waist circumference = 100.6 cm) and decreased grip strength (5% or greater decrease in maximum grip strength) [31]. In this longitudinal study, higher fat mass percent and greater waist circumference were marginally associated with prevalent UI at least monthly, but strongly associated with prevalent UI at least weekly [31]. An association between UUI and medical factors/diseases (diabetes, heart disease, anxiety, depression, constipation, and brain injury) was found in a recent community-based study [32]. Gerst et al*.* found that prostate problems (unspecified), a higher number of comorbid conditions (mean number of chronic conditions = 2.5) and other factors such as limitations in Activities of Daily Living (ADL) were significant independent predictors of UUI [65]. UUI was also linked to frailty, faecal incontinence and depressive symptoms [67]. Increased BMI/overweight/obesity was a common medical factor in community-based studies [31, 32, 45–47, 68], whereas hypertension was less common [32, 68]. Tsui et al*.* identified increased BMI and high blood pressure as vascular risk factors for UUI [68]. At 43%, UUI was the most frequently documented type of established UI in this review [31–33, 40, 42, 46–48, 51, 54, 59, 65, 67–69, 72, 73, 75]. Detrusor overactivity (DO) ranked first in the other medically-related risk factors category/third overall [35, 39, 40, 51, 54, 55, 57, 71, 72, 74] and was associated with UUI [40, 51, 72] and nocturnal enuresis [39].

Identified factors associated with SUI included Diabetes mellitus [42, 46], heart disease (unspecified), poor vision and faecal incontinence [69].

Increased BMI [46, 47], increasing age [46], and other factors including poor physical function (indicated by lower Activities of Daily Living Scale (ADLS) scores) and poor sleep quality (higher Pittsburgh Sleep Quality Index (PSQI) scores) were also associated with SUI [69].

Four included studies focused exclusively on nursing homes (NH) [34, 35, 61, 64], and documented environmental risk factors such as poor lighting, cold weather, lack of commodes, use of cot-sides/bedrails, reliance on draw sheets and pads, and forgotten call bells [35]. UI was also correlated with medical factors/diseases including Dementia [34, 35], UTI, BPH/bladder stones, Stroke, Parkinson’s disease (PD), faecal impaction [35], and depressive symptoms [64]. Race, as a demographic factor [61], was reported along with other factors like medications [35], poor physical function and poor cognitive status [64]. African-Americans, especially African-American men, had higher UI odds [61]. The development of nocturnal enuresis was associated with age-related physiological changes related to detrusor instability/overactivity, reduction in functional bladder capacity, increased post-void residual volume, renal function decline, increased night-time urine production, decreased awareness of bladder filling, and decreased bladder emptying efficiency [35]. Diabetes was frequently correlated with UI in geriatric care facilities [42].

Studies in tertiary healthcare facilities identified behavioural, demographic [49], disease-related [49, 52], and other factors [50]. These are detailed below in relation to their corresponding patient characteristics.

### UI risk factors and population characteristics

Among older men with frailty, Landi et al*.* found that urinary tract infection, physical restraints, and environmental barriers were potentially reversible risk factors. Non-reversible UI risk factors included advanced age, physical limitations, cognitive impairment, and diabetes mellitus [58]. Diabetes was also a primary risk factor for urinary and faecal incontinence among the oldest old men in a Canadian longitudinal study [76].

Among men with BPH, Prada et al*.* identified tobacco smoking and alcohol use, urban dwelling and occupation (work requiring a high degree of physical effort and jobs that require sitting for longer periods), and medical/disease-related factors, such as Heart failure, Diabetes mellitus and having at least three other comorbidities [49].

Among men following post-robotic radical prostatectomy, myosteatosis (low average total psoas density), low obturator internus muscle thickness and short membranous urethral length were recently reported by Yamashita et al*.*, with myosteatosis being considered a novel predictor of post-prostatectomy incontinence [50]. In a cohort of older men with SUI, the majority of whom were offered prostatectomy for prostate cancer, male SUI was associated with multi-morbidity, functional dependence, and frailty [77].

Among men undergoing artificial urethral sphincter (AUS) placement post-prostatectomy, low serum testosterone was reported as a risk factor for stress UI [44]. In community-dwelling older male cancer patients, diagnoses of prostate and bladder cancers had the strongest associations with UI, compared to colorectal and lung cancers [43]. Furthermore, Kopp et al. identified prostatectomy, post-radiation therapy, observation/watchful waiting in prostate cancer (Ca) and androgen deprivation therapy for prostate Ca as risk factors for post-prostatectomy incontinence among elderly prostate Ca survivors [66]. Moore also described prostatectomy-related neural injury, ischemia during surgery, scar tissue immobilizing the sphincter, short membranous urethral length, surgical technique and preoperative radiotherapy as causative factors for post-prostatectomy incontinence along with increasing age [74].

Among relatively healthy older men, Cheng et al*.* showed a causal relationship between UI and increasing age, as well as diabetes mellitus [46].

## Discussion

In this scoping review, evidence in all contexts was systematically synthesised in relation to older men. Despite the lack of systematically conducted reviews identifying and categorising UI risk factors in older men, we found systematic reviews on UI in nursing home residents as well as on male UI risk factors in general, although uncategorised, with which to compare our findings [3, 78]. Our findings revealed that behavioural risk factors were the least investigated. Lifestyle factors including sedentary behaviour are rarely the focus of UI research [79]. In an effort to fill the evidence gap, Farrés-Godayol and colleagues found that nursing home residents with UI spent significantly more sedentary time compared to continent residents [79]. According to the Seventh International Consultation on Incontinence, rigorous studies on lifestyle interventions are needed [80]. This emphasises the need for more research that explores the breadth of lifestyle/behavioural factors to inform lifestyle interventions.

Age was the top risk factor, consistent with findings from a systematic review of UI and associated risk factors in nursing home residents, which found that age and sex were the most frequently studied risk factors out of 45 from 16 studies [78]. The pooled prevalence of UI increased with age and functional dependency [3]. Many medical conditions have been implicated in the multifactorial aetiology of UI in older adults [40]. Similar to our findings, an association was found between BPH and UI in men aged 60 + [81]. A systematic review also reported associations between UI in community-dwelling men and stroke, diabetes, poor general health, radiation, and prostate cancer surgery, although age groups were not specified [3]. The European Patients’ Academy on Therapeutic Innovation noted the importance of the coexistence of multiple factors, an understanding of which is crucial to evaluating patients and developing relevant interventions for older adults with urinary incontinence effectively [24].

Studies show that DO is the most common cystometric abnormality in patients with PD and is one of the most common forms of urinary dysfunction in people with idiopathic PD (IPD) [82, 83]. The finding of ADL disability is consistent with reports on Hispanic longitudinal data from community-dwelling older adults, which included functional impairment and ageing as risk factors for incident incontinence [84]. A bidirectional relationship between functional decline/disability and UI was described by Coll-Planas et al., in which continence reduction leads to functional decline, and functional decline leads to further continence decline [85]. In other studies, obesity was associated with an increased odds of UI [86]. Depression increased the odds of UI among elderly people in the Brazilian SABE study, and UI prevalence was higher when there was high physical dependence [87]. According to a German UI survey, five or more comorbid conditions increased incontinence risk to 100% [88].

Institutionalisation increased UI prevalence following facility admission [72]. For NH, urinary incontinence is a quality of care indicator and a high prevalence of UI is often regarded as a sign of poor quality care [61, 89].

### Strengths and limitations

This scoping review focused on the collation, identification and categorisation of risk factors for UI in older men by mapping and synthesising the breadth of evidence and identifying knowledge gaps in a systematic and comprehensive manner. An exhaustive search across all sources was conducted that produced robust evidence. Study type or publication date was not restricted, and there was no language restriction.

The lack of age stratification in most data on men in general and the paucity of data specifically on older men limited the amount of eligible evidence sources.

## Conclusions

This scoping review found limited evidence on factors, other than those related to medical diagnoses, that might contribute to UI in older men. Available data are limited by the inability to extract data specific to older men, rather than men or older adults in general. The primary evidence sources originated from only 6% of countries in the world, making generalisations difficult.

### Recommendations for future studies

There is a need for more primary research focusing on behavioural risk factors for UI in older men due to the lack of evidence on this topic. These factors may play a role in health promotion and disease prevention in this area. It is imperative that more UI research be conducted in areas where there are no existing data on the topic. More research should be encouraged in primary care settings since primary care is the first point of care for the vast majority of patients.

### Supplementary Information

**Additional file 1:** APPENDICES: Search strategies, data extraction form and PRISMA-ScR checklist.

## Data Availability

A reasonable request may be made to the corresponding author to obtain the dataset used in this study.
